# A Two-Stage Exon Recognition Model Based on Synergetic Neural Network

**DOI:** 10.1155/2014/503132

**Published:** 2014-03-25

**Authors:** Zhehuang Huang, Yidong Chen

**Affiliations:** ^1^School of Mathematics Sciences, Huaqiao University, Quanzhou 362021, China; ^2^Cognitive Science Department, Xiamen University, Xiamen 361005, China; ^3^Fujian Key Laboratory of the Brain-Like Intelligent Systems, Xiamen 361005, China

## Abstract

Exon recognition is a fundamental task in bioinformatics to identify the exons of DNA sequence. Currently, exon recognition algorithms based on digital signal processing techniques have been widely used. Unfortunately, these methods require many calculations, resulting in low recognition efficiency. In order to overcome this limitation, a two-stage exon recognition model is proposed and implemented in this paper. There are three main works. Firstly, we use synergetic neural network to rapidly determine initial exon intervals. Secondly, adaptive sliding window is used to accurately discriminate the final exon intervals. Finally, parameter optimization based on artificial fish swarm algorithm is used to determine different species thresholds and corresponding adjustment parameters of adaptive windows. Experimental results show that the proposed model has better performance for exon recognition and provides a practical solution and a promising future for other recognition tasks.

## 1. Introduction

With the completion of human genome project, gene data increase exponentially. Identifying the genes encoding of DNA [1] has important theoretical and practical implications. How to quickly access accurate genetic information is an urgent problem to be solved.

Early exon recognition methods were based mainly on statistical models [2], which get their chromosomal order by statistical analysis of different genes. But with the increase of genomic number, statistical methods cannot meet the need for rapid recognition of exons. At present, exon recognition methods based on digital signal processing have also been widely used [3–5]. These techniques select a suitable mapping method and transformation method to get spectral values and identify exons according to fixed length window. Limitations of these methods include slow recognition speed and inability to accurately determine the threshold for different species.

Synergetic theory [6] is the science proposed by Haken to describe high dimension and nonlinear problem as a set of low-dimension nonlinear equations. One advantage of synergetic neural network is that the method is robust against noise and the method can better handle the fuzzy matching problem [7–9]. Exon recognition can also be considered a problem of pattern recognition, for which the proposed method can be used to solve.

Artificial fish swarm algorithm (AFSA) [10, 11] is a class of swarm intelligence optimization algorithms based on the behavior of animals proposed in 2002; the basic idea of AFSA is to imitate the fish behaviors such as praying, swarming, and following. AFSA is very suitable for solving a variety of numerical optimization problems, making the algorithm become a hot topic in the current optimization field quickly. Because of simplicity in principle and good robustness, AFSA has been applied successfully to all kinds of optimization problems such as image segmentation [12], color quantization [13], neural network [14], fuzzy logic controller [15], multirobot task scheduling [16], fault diagnosis in mine hoist [17], data clustering [18], and other areas.

In this paper, we proposed a two-stage exon recognition model based on synergetic neural network and artificial fish swarm algorithm. This paper is organized as follows. Firstly, traditional exon recognition method based on digital signal processing and related work are presented. Secondly, an exon recognition model based on synergetic neural network and parameter optimization method based on artificial fish swarm algorithm are introduced. Finally some experimental tests, results, and conclusions are given on the systems.

## 2. Introduction to Exon Recognition Method Based on Digital Signal Processing

**Figure 1:**
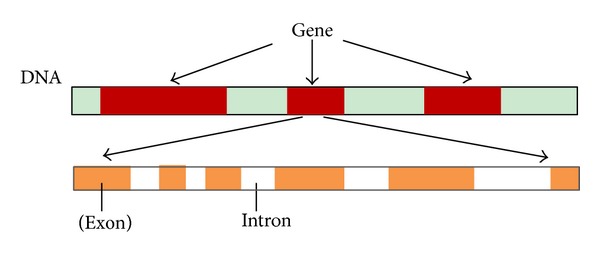
Structure of eukaryotic DNA sequence.

The gene is usually divided into many fragments. The coding sequence is called exons and noncoding part is called introns, as shown in Figure 1.

**Figure 2:**
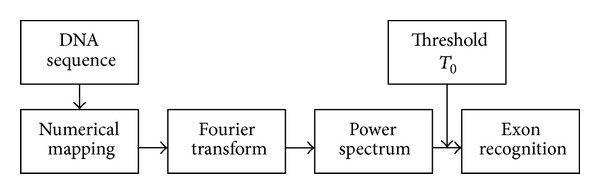
Exon recognition algorithm based on 3-Cycle spectrum.

The objective of gene recognition is to identify the exons of DNA sequence. Gene recognition based on digital signal processing methods consists of several steps [19, 20]. First, gene sequences are transformed into digital symbol sequences using mapping methods [21–24]. This is followed by calculation of the corresponding frequency value by fast Fourier transform and the 3-Cycle properties of the spectrum are then used to identify exons [25, 26]. Finally, fixed sliding window method is used for automatic exon recognition, as shown in Figure 2.

### 2.1. Z-Curve Mapping

In order to make digital processing, we must transform four nucleotide sequences A, T, G, and C into their corresponding numeric sequence based on certain rules.

Let the four instruction sequences be {*u*_*b*_[*n*]}, *b* ∈ *I* = {A, C, G, T}, and cumulative sequence *b*_*n*_  (*n* = 0,1 …, *N* − 1) is *b*_*n*_ = ∑_*i*=0_^*n*−1^ *u*_*b*_[*i*]; then we can define three sequences *x*[*n*], *y*[*n*], and *z*[*n*]:

(1) $$\begin{array}{c} x\left[n\right]=2\left({\text{A}}_{n}+{\text{G}}_{n}\right)-n,\\ y\left[n\right]=2\left({\text{A}}_{n}+{\text{C}}_{n}\right)-n,\\ z\left[n\right]=2\left({\text{A}}_{n}+{\text{T}}_{n}\right)-n. \end{array}$$

Let

(2) $$\begin{array}{c} x\left[-1\right]=0,\;\;y\left[-1\right]=0,\;\;z\left[-1\right]=0,\\ \Delta x\left[n\right]=x\left[n\right]-x\left[n-1\right],\;\;\Delta y\left[n\right]=y\left[n\right]-y\left[n-1\right],\\ \Delta z\left[n\right]=z\left[n\right]-z\left[n-1\right]. \end{array}$$

Thus we can get the *Z*-curve mapping:

(3) $$\begin{array}{c} \left(\begin{array}{c} \Delta x\left[n\right]\\ \Delta y\left[n\right]\\ \Delta z\left[n\right] \end{array}\right)=\left(\begin{array}{cccc} 1 & -1 & 1 & -1\\ 1 & 1 & -1 & -1\\ 1 & -1 & -1 & 1 \end{array}\right)\left(\begin{array}{c} u_{\text{A}}\left[n\right]\\ u_{\text{C}}\left[n\right]\\ u_{\text{G}}\left[n\right]\\ u_{\text{T}}\left[n\right] \end{array}\right). \end{array}$$

For example, the DNA sequence of *S*(*n*) is ACGTTAG; then the corresponding *Z*-curve mapping sequence is shown in Table 1.

### 2.2. The Power Spectrum

To study the characteristics of DNA coding sequences (exons), we can do the discrete Fourier transform (DFT), respectively, for the instruction sequences:

(4) $$\begin{array}{c} U_{b}\left[k\right]=\sum_{n=0}^{N-1}u_{b}\left[n\right]e^{-j(2\pi nk/N)},\;k=0,1,\ldots ,N-1. \end{array}$$

Thus we can calculate the power spectrum:

(5) Pz[k]=|ΔX[k]|2+||ΔY[k]||2+|ΔZ[k]|2,    k=0,1,…,N−1,

where Δ*X*[*k*], Δ*Y*[*k*], and Δ*Z*[*k*] are the Fourier transform of Δ*x*[*n*], Δ*y*[*n*], and Δ*z*[*n*], respectively.

The spectral peaks of exon sequences are larger in *k* = *N*/3 and *k* = 2*N*/3 of the power spectrum curve, while they are not similar for intron. This statistical phenomenon is known as 3-Cycle. Suppose that the average power spectrum of DNA sequences is

(6) $$\begin{array}{c} \overset{-}{E}=\frac{\sum_{k=0}^{N-1}P\left[k\right]}{N}. \end{array}$$

The power spectrum ratio of the DNA sequence and the average spectrum of the entire sequence are known as SNR (signal-to-noise ratio):

(7) $$\begin{array}{c} R=\frac{P\left[N/3\right]}{\overset{-}{E}}. \end{array}$$

**Figure 3:**
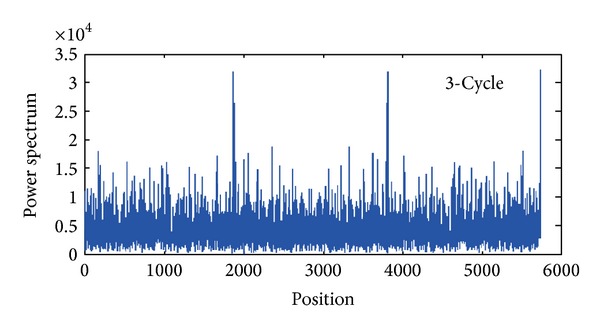
The power spectrum of viral gene sequence.

Figure 3 shows the power spectrum of viral genes.

From Figure 3, we can see that the spectrum presents obvious 3-Cycle. The peaks appear roughly in 2000, 4000, and 6000. So the exon segment can be determined, enabling the recognition of genes.

The highest point of power spectrum may not appear in *k* = *N*/3 and *k* = 2*N*/3 but occur in the surrounding. So we can calculate average SNR *R*_1_ and *R*_2_ of intervals [*N*/3 − *γ*, *N*/3 + *γ*] and [2*N*/3 − *γ*, 2*N*/3 + *γ*], respectively:

(8) $$\begin{array}{c} R_{1}=\frac{\sum_{k=N/3-\gamma }^{N/3+\gamma }P\left[E\right]}{\left(2\gamma +1\right)\overset{-}{E}},\;\;R_{2}=\frac{\sum_{k=2N/3-\gamma }^{2N/3+\gamma }P\left[E\right]}{\left(2\gamma +1\right)\overset{-}{E}}. \end{array}$$

### 2.3. Automatic Recognition Algorithm Based on Fixed Sliding Windows

Supposed *M* is the length of fixed window; we can do four discrete Fourier transforms (DFT) for instruction sequences {*u*_*b*_[*n*]}  (0 ≤ *n* ≤ *N* − 1),

(9) $$\begin{array}{c} U_{b}\left[k\right]=\sum_{i=n-\left(M-1\right)/2}^{i=n+\left(M-1\right)/2}u_{b}\left[i\right]e^{-j(2\pi ik/M)},\;k=0,1,\ldots ,M-1. \end{array}$$

Then the total spectrum *p*  (*n*; *M*/3) at position *M*/3 is

(10) P[M3]=|UA[M3]|2+|UT[M3]|2 +|UG[M3]|2+|UC[M3]|2=Δp(n;M3).

## 3. Related Work

The SNR of exon sequences reflects the distribution of spectrum peak. SNR greater than a given threshold is a characteristic of exons, while introns generally do not have this property.

Protein coding regions and noncoding regions can be distinguished using the value of SNR, but this method still has a large predictive error because the spectrum peak varies amongst different biological categories. A fixed threshold is unreasonable to use for different biological categories. Therefore, determining the SNR threshold has great significance for exon recognition. Note that it is difficult to find the proper prediction threshold for biological categories when relying only on prior biological knowledge.

Xu [27] proposed a method based on bootstrap algorithm to determine the best SNR threshold that can be obtained from marked exon sequences. The results of that study showed that the average prediction accuracy of the method was 81%, which is 19% higher than other methods that employ empirical thresholds. In paper [28], a novel model was proposed to determine the SNR threshold based on the means of biological categories and improved the recognition performance to some extent.

But all the methods mentioned above have problems, such as slow recognition speed, inaccurate determination of the threshold for different species, and the requirement to know the exon fragments of DNA sequences. In the following sections, we propose a novel two-stage exon recognition model based on synergetic neural network and artificial fish swarm algorithm to better deal with these problems.

## 4. A Novel Two-Stage Exon Recognition Model

In this section, a two-stage exon recognition model is presented. In the first stage, synergetic neural network is used to determine initial exon intervals. In the second stage, final accurate exon intervals determination based on adaptive sliding window and parameter optimization algorithm are introduced.

### 4.1. Initial Exon Intervals Determination Based on Synergetic Neural Network

The basic principle of synergetic neural network [29, 30] is that the pattern recognition procedure can be viewed as the competition progress of many order parameters. The strongest order parameter will win by competition and desired pattern will be recognized.

A pattern that remained to be recognized, *q*, is constructed by a dynamic process which translates *q* into one of prototype pattern vectors *v*_*k*_ through status *q*(*t*); namely, this prototype pattern is closest to *q*(0). The process is described as the following equation:

(11) $$\begin{array}{c} q⟶q\left(t\right)⟶v_{k}. \end{array}$$

A dynamic equation can be given for an unrecognized pattern *q*:

(12) q˙=∑k=1Mλkvk(vk+q)−B∑k′≠k(vk′+q)2(vk+q)vk −C(q+q)q+F(t),

where *q* is the status vector of input pattern with initial value *q*_0_, *λ*_*k*_ is attention parameter, *v*_*k*_ is prototype pattern vector, and *v*_*k*_^+^ is the adjoint vector of *v*_*k*_ that satisfies

(13) $$\begin{array}{c} \left(v_{k}^{+},v_{k^{\prime }}^{\text{T}}\right)=v_{k}^{+}\cdot v_{k^{\prime }}^{\text{T}}=\delta_{kk^{\prime }}. \end{array}$$

Corresponding dynamic equation of order parameters is

(14) $$\begin{array}{c} {\dot{\xi }}_{k}=\lambda_{k}\xi_{k}-B\sum_{k^{\prime }\neq k}\xi_{k^{\prime }}^{2}\xi_{k}-\text{C}\left|\sum_{k^{\prime }=1}^{M}\xi_{k^{\prime }}^{2}\right|\xi_{k}. \end{array}$$

Haken has proved that when *λ*_*k*_ = *c* (*c* > 0), the largest initial order parameter will win and the network will then converge.

**Figure 4:**
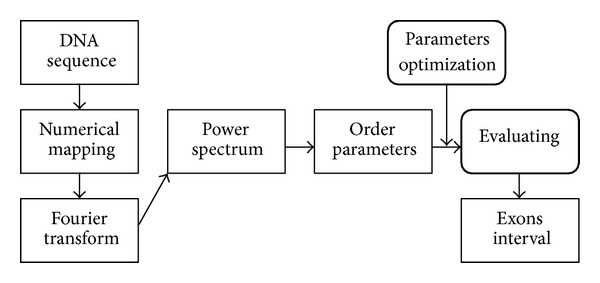
Exon recognition based on synergetic neural network.

We firstly introduce the synergetic theory to exon recognition; an exon recognition algorithm based on synergetic neural network is shown in Figure 4.

We use synergetic neural network and *N* equal method to quickly determine the initial exon region, as shown in Algorithm 1.

### 4.2. Get Precise Exon Intervals Using Adaptive Smoothing Window

We can obtain several possible exon intervals by Algorithm 1. In this section, we propose an adaptive sliding window algorithm to get more accurate intervals, as shown in Algorithm 2.

### 4.3. Parameter Optimization Based on Artificial Fish Swarm Algorithm

The parameters T_0_ and *γ* directly influence the performance of exon recognition. The adjustment of the parameters is a global behaviour and has no general research theory to control the parameters in the recognition process at present. In this section, artificial fish swarm algorithm is used to search the global optimum parameters (T_0_, *γ*) in the corresponding parameter space.

The parameter optimization based on artificial fish swarm algorithm is shown as Algorithm 3.

## 5. Experiment

### 5.1. Data Description

In our experiments, we use some gene sequences provided by Chinese Graduate Mathematical Contest in Modeling. Chinese graduate Mathematical Contest in modeling is aimed at improving the students' comprehensive abilities of mathematical modeling and computer to solve practical problems. From different points of view, the integrated use of a variety of mathematical methods established the mathematical model of the characteristic.

We selected 100 human gene sequences, 100 rodent gene sequences (including* Mus musculus* and Sewer rat), and 200 mammalian gene sequences for testing. The signal-to-noise ratios of the sequences are gotten by SPSS statistical analysis software, as shown in Table 2.

From Table 2, we can find out that the difference between SNR standard deviation of exons is greater than SNR standard deviation of introns.

**Figure 5:**
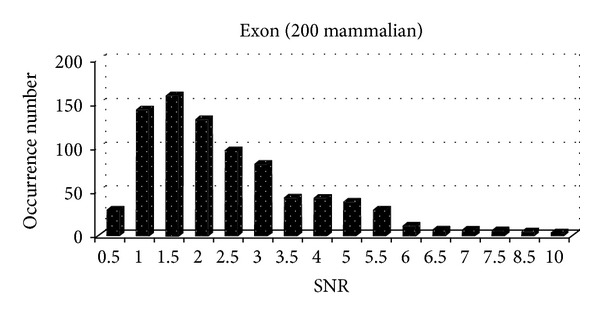
The SNR distribution of 200 mammalian exons.

**Figure 6:**
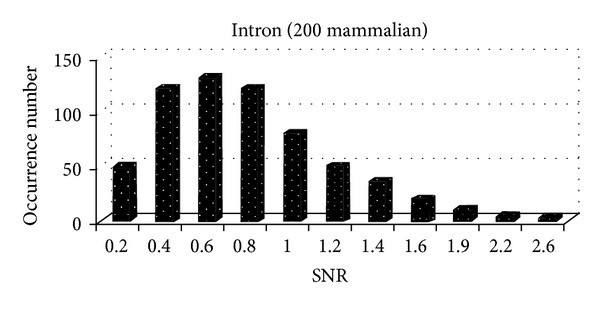
The SNR distribution of 200 mammalian introns.

At the same time, we analyze the SNR distribution of exons and introns of 200 mammalian gene sequences, as shown in Figure 5 and Figure 6.

From Figure 5 and Figure 6, we can see that the mammalian introns are mostly less than 2, while exons are mostly distributed in the range of [0,2], which accounts for 55.38%. Therefore, it is unreasonable to set SNR threshold of different categories as fixed value. How to accurately determine SNR threshold of each kind of biological gene has important significance.

### 5.2. Experiment Results

Suppose that sensitivity *S*_*N*_ = *T*_*P*_/(*T*_*p*_ + *F*_*N*_) and specificity *S*_*P*_ = *T*_*N*_/(*T*_*N*_ + *F*_*P*_), where *T*_*P*_ is the number of exons which are correctly identified, *T*_*N*_ is the number of introns which are correctly identified, *F*_*P*_ is the number of exons which are not correctly identified, and *F*_*N*_ is the number of introns which are not correctly identified. Then we can compute the accurate rate A_*c*_ = (*S*_*N*_ + *S*_*P*_)/2.

For comparison, we use four strategies.

1. Baseline: automatic recognition algorithm with threshold *R*_0_ = 2.
2. Bootstrap: the threshold selection algorithm based on bootstrap method.
3. SNN: exon recognition based on synergetic neural network.
4. SNN + AFSA: two-stage exon recognition model based on synergetic neural network and artificial fish swarm algorithm.

The testing performance of Baseline is shown as in Table 3.

The experiments showed that when the exon length is short, the recognition accuracy rate is low. In the short gene coding sequence, 3-base periodicity is not absolutely satisfied.

In our experiments, we complete a two-stage exon recognition model based on synergetic neural network and artificial fish swarm algorithm. The parameter settings of artificial fish swarm algorithm are shown in Table 4.

In the experiment, we set the recognition accuracy rate as score function.

The testing performance of SNN + AFSA is shown as in Table 5.

Table 5 shows that the two-stage exon recognition algorithm improves precision compared to the Baseline system. Experiments also indicate that the improved model has a more powerful global exploration ability and a reasonable convergence speed.

The accurate rate A_*c*_ of different methods is shown in Table 6.

Detailed comparisons of results are given in Table 6. Experimental results show that the proposed model SNN and SNN + AFSA have good performance for exon recognition. The accurate rate we obtained for all four corpuses is comparable to the state-of-the-art systems, such as Baseline and bootstrap method. Through the evaluating of order parameter equation of SNN to obtain the best threshold, we can further improve the exon recognition performance.

At the same time, we can see that the performance of SNN + AFSA is better than SNN model. This is because the attention parameters are very important for SNN and optimization algorithm is essential for better performance. Experimental results show that improved AFSA algorithm has better global and local parameter searching capabilities and thus a better recognition result.

It is worth noting that experimental results show that run times of our proposed model reduced with good speedup ratio compared with Baseline. Further studies show that the procedure exhibits data parallelism, so it can be effectively parallelized by running it concurrently. In the future work, we will utilize parallel processing techniques for rapid exon recognition based on SNN to further reduce the run time.

## 6. Conclusions

In the paper, we proposed a two-stage exon recognition model based on synergetic neural network and artificial fish swarm algorithm. Experiments show that the proposed model can improve the precision of exon recognition.

We got the following conclusions.

1. The exon recognition procedure can be viewed as the competition progress of many order parameters. The proposed model based on synergetic neural network and *N* equal method can quickly determine the exon intervals.
2. Artificial fish swarm algorithm has both global and local search ability and can effectively choose the parameters of our proposed model.
3. Using *N* equal algorithm to obtain exon intervals may still miss some intervals which are in the middle; we will further improve the algorithm or use different pattern recognition algorithm in the future.

It must be noted that, although we have made some efforts to explore the intelligent exon recognition algorithm in this paper. But due to the special nature of life science itself, there are many problems such as how to accurately determine that the exon interval needs further study. But we believe that with the development of social progress and technology, gene identification technology will become increasingly perfect; we expect it can bring gospel to manking in the near future.

## Figures and Tables

**Algorithm 1:**
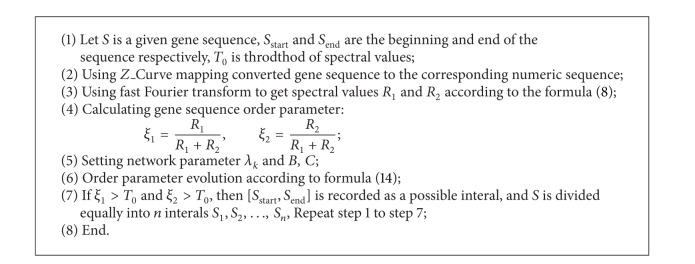
Determination of initial exon region based on synergetic neural network.

**Algorithm 2:**
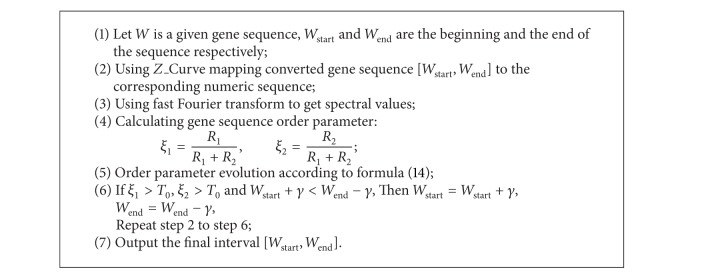
Precise exon regions based on adaptive smoothing window.

**Algorithm 3:**
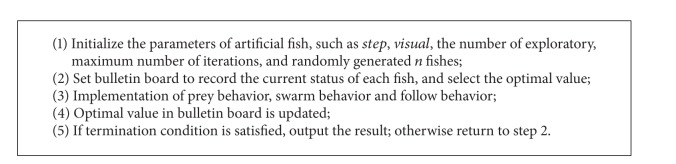
Parameter optimization based on artificial fish swarm algorithm.

**Table 1:** 

Δ*x*[*n*]	1	−1	1	−1	−1	1	1
Δ*y*[*n*]	1	1	−1	−1	−1	1	−1
Δ*z*[*n*]	1	−1	−1	1	1	1	−1

**Table 2:** The signal-to-noise ratio of four different gene sequences.

Gene categories	Exon			Intron		
	Number	*R*-mean	Variance	Number	*R*-mean	Variance
Human	35	3.02	3.071	26	0.82	0.533
*Mus musculus *	357	2.46	2.508	275	0.68	0.414
Sewer rat	45	3	5.233	35	0.83	0.624
Mammalian	827	2.72	6.243	626	0.67	0.394

**Table 3:** The testing performance of Baseline.

Gene categories	*T*_*P*_	*F*_*N*_	*S*_*N*_	*T*_*N*_	*F*_*P*_	*S*_*P*_	*A*_*c*_
Human	17	18	0.485	24	2	0.923	0.71
*Mus musculus *	146	211	0.409	271	4	0.985	0.70
Sewer rat	17	28	0.378	31	4	0.886	0.63
Mammalian	369	458	0.446	621	5	0.992	0.72

**Table 4:** The parameter settings of artificial fish swarm algorithm.

Algorithm	Fish number	Visual	Delta	Step	Number of iterations
AFSA	100	2.85	9	1	60

**Table 5:** The test performance of SNN + AFSA.

Gene categories	*T*_*P*_	*F*_*N*_	*S*_*N*_	*T*_*N*_	*F*_*P*_	*S*_*P*_	*A*_*c*_
Human	30	5	0.857	19	7	0.731	0.79
* Mus musculus *	295	62	0.826	220	55	0.80	0.81
Sewer rat	36	9	0.80	28	7	0.80	0.80
Mammalian	630	197	0.762	607	19	0.97	0.87

**Table 6:** The test performance comparison among different methods.

Gene categories	Baseline	Bootstrap	SNN	SNN + AFSA
Human	0.71	0.76	0.78	0.79
*Mus musculus *	0.70	0.78	0.80	0.81
Sewer rat	0.63	0.75	0.77	0.80
Mammalian	0.72	0.84	0.85	0.87

## References

[1] Burge CB, Karlin S (1998). Finding the genes in genomic DNA. *Current Opinion in Structural Biology*.

[2] Wang Z, Chen Y, Li Y (2004). A brief review of computational gene prediction methods. *Genomics Proteomics Bioinformatics*.

[3] Sharma SD, Shakya K, Sharma SN Evaluation of DNA mapping schemes for exon detection.

[4] Kotlar D, Lavner Y (2003). Gene prediction by spectral rotation measure: a new method for identifying protein-coding regions. *Genome Research*.

[5] Yin C, Yau SS-T (2007). Prediction of protein coding regions by the 3-base periodicity analysis of a DNA sequence. *Journal of Theoretical Biology*.

[6] Haken H (1991). *Synergetic Computers and Cognition-A Top-Down Approach to Neural Nets*.

[7] Shao J, Gao J, Yang XZ Synergetic face recognition algorithm based on ICA.

[8] Jiang Z, Dougal RA (2004). Synergetic contro1 of power converters for pulse current charging of advanced batteries from a fuel cell power source. *IEEE Transactions on Power Electronics*.

[9] Ma XL, Jiao LC, Campilho A, Kamel M (2004). Reconstruction of order parameters based on immunity clonal strategy for image classification. *Image Analysis and Recognition*.

[10] Li XL, Feng SH, Qian JX, Lu F (2004). Parameter tuning method of robust pID controller based on artificial fish school algorithm. *Chinese Journal of Information and Control*.

[11] Li XL, Lu F, Tian GH, Qian JX (2004). Applications of artificial fish school algorithm in combinatorial optimization problems. *Chinese Journal of Shandong University: Engineering Science*.

[12] Tian W, Geng Y, Liu J, Ai L Optimal parameter algorithm for image segmentation.

[13] Yazdani D, Nabizadeh H, Kosari EM, Toosi AN Color quantization using modified artificial fish swarm algorithm.

[14] Zhang M, Shao C, Li F, Gan Y, Sun J Evolving neural network classifiers and feature subset using artificial fish swarm.

[15] Chen D, Shao L, Zhang Z, Yu X An image reconstruction algorithm based on artificial fish-swarm for electrical capacitance tomography system.

[16] Wang C-J, Xia S-X (2010). Application of probabilistic causal-effect model based artificial fish-swarm algorithm for fault diagnosis in mine hoist. *Journal of Software*.

[17] Tian W, Tian Y, Ai L, Liu J A new optimization algorithm for fuzzy set design.

[18] Cheng Y, Jiang M, Yuan D Novel clustering algorithms based on improved artificial fish swarm algorithm.

[19] Berryman MJ, Allison A, Wilkinson CR, Abbott D (2005). Review of signal processing in genetics. *Fluctuation and Noise Letters*.

[20] von Öhsen N, Sommer I, Zimmer R, Lengauer T (2004). Arby: automatic protein structure prediction using profile-profile alignment and confidence measures. *Bioinformatics*.

[21] Kotlar D, Lavner Y (2003). Gene prediction by spectral rotation measure: a new method for identifying protein-coding regions. *Genome Research*.

[22] Yin C, Yau SS-T (2007). Prediction of protein coding regions by the 3-base periodicity analysis of a DNA sequence. *Journal of Theoretical Biology*.

[23] Rushdi A, Tuqan J Gene identification using the stroke Z sign-curve representation.

[24] Sharma SD, Shakya K, Sharma SN Evaluation of DNA mapping schemes for exon detection.

[25] Kotlar D, Lavner Y (2003). Gene prediction by spectral rotation measure: a new method for identifying protein-coding regions. *Genome Research*.

[26] Bo L, Ding K (2005). Graphical approach to analyzing DNA sequences. *Journal of Computational Chemistry*.

[27] Xu SL (2011). *Threshold selection of gene prediction Based on Bootstrap algorithm [M.S. thesis]*.

[28] Shao JF, Yan XH, Shao S (2013). SNR of DNA sequences mapped by general affine transformations of the indicator sequences. *Journal of Mathematical Biology*.

[29] Jiang Z, Dougal RA (2004). Synergetic contro1 of power converters for pulse current charging of advanced batteries from a fuel cell power source. *IEEE Transactions on Power Electronics*.

[30] Gao J, Dong H, Shao J, Zhao J (2005). Parameters optimization of synergetic recognition approach. *Chinese Journal of Electronics*.

